# Development of an Environmental Impact Calculator for Minimally Invasive Cholecystectomy: Protocol and Methodological Framework of the ECOMIS Project

**DOI:** 10.3390/healthcare14213622

**Published:** 2026-10-26

**Authors:** Hidde C. G. Overtoom, Mark Portelli, Sergi Sanchez-Cordero, Jada Saunders, Natalia Dowgiałło-Gornowicz, Myrthe M. M. Eussen, Stefania Marconi, Lisa H. Massey, Barbara Seeliger, Patricia Sylla, Marc G. H. Besselink, Nicole D. Bouvy, Mark I. van Berge Henegouwen, Suzanne S. Gisbertz

**Affiliations:** 1 Department of Surgery, Amsterdam UMC, Location University of Amsterdam, 1081 HV Amsterdam, The Netherlands; h.c.g.overtoom@amsterdamumc.nl (H.C.G.O.); m.g.besselink@amsterdamumc.nl (M.G.H.B.); m.i.vanbergehenegouwen@amsterdamumc.nl (M.I.v.B.H.); s.s.gisbertz@amsterdamumc.nl (S.S.G.); 2 Cancer Treatment and Quality of Life, Cancer Center Amsterdam, 1081 BT Amsterdam, The Netherlands; 3 Amsterdam Gastroenterology Endocrinology Metabolism, Amsterdam UMC, University of Amsterdam, 1012 WX Amsterdam, The Netherlands; 4 Department of General and Vascular Surgery, Mater dei Hospital, Tal-Qroqq, MSD 2090 Msida, Malta; mark.c.portelli@gov.mt; 5 Department of Metabolic and Bariatric Surgery, University Hospital Moises Broggi, 08970 Sant Joan Despí, Barcelona, Spain; sergi.sanchezcordero@gmail.com; 6 Department of General Surgery, County Durham and Darlington NHS Foundation Trust Durham, Darlington DL3 6HX, UK; jae.marie95@hotmail.com; 7 Department of General, Minimally Invasive, and Elderly Surgery, University of Warmia and Mazury, 10-719 Olsztyn, Poland; 8 Department of Surgery, Maastricht University Medical Center, 6229 HX Maastricht, The Netherlands; myrthe.eussen@mumc.nl (M.M.M.E.); n.d.bouvy@lumc.nl (N.D.B.); 9 NUTRIM School of Nutrition and Translational Research in Metabolism, Maastricht University, 6200 MD Maastricht, The Netherlands; 10 Department of Civil Engineering and Architecture, University of Pavia, 27100 Pavia, Italy; stefania.marconi@unipv.it; 11 Department of Colorectal Surgery, Nottingham University Hospitals NHS Trust, Nottingham NG5 1PB, UK; lisahmassey@gmail.com; 12 IHU Strasbourg, Institute of Image-Guided Surgery, 67091 Strasbourg, France; barbara.seeliger@ihu-strasbourg.eu; 13 Department of Digestive and Endocrine Surgery, University Hospitals of Strasbourg, 67000 Strasbourg, France; 14 ICube, UMR 7357, CNRS, INSERM U1328 RODIN, University of Strasbourg, 67081 Strasbourg, France; 15 Department of Surgery, Division of Colon and Rectal Surgery, Mount Sinai Health System, New York, NY 10029, USA; patricia.sylla@mountsinai.org

**Keywords:** minimally invasive surgery, environmental impact, calculator, tailor-made advice

## Abstract

**Highlights:**

**What are the main findings?**

• ECOMIS will develop a standardized tool to assess the environmental impact of minimally invasive cholecystectomy.

**What are the implications of the main findings?**

• It may help identify modifiable sources of environmental burden in surgery.
• It may support more sustainable surgical practice.

**Abstract:**

One of the greatest threats to public health in the 21st century is anthropogenic climate change. Healthcare services worldwide account for 4.9% of global CO_2_ emissions, with operating rooms being the most resource-intensive areas of the hospital. The need for environmentally sustainable change is a priority. Reliable quantitative data on emissions remain limited, which may hinder informed decision-making on surgical sustainability. The aim of this study is to develop and validate an easily accessible and user-friendly calculator to quantitatively estimate procedure-related carbon dioxide equivalent (CO_2_-eq) emissions of minimally invasive cholecystectomy across a range of countries, hospital volumes, and healthcare settings. The ECOMIS project follows a multiphase, stepwise design. The present study will focus on minimally invasive cholecystectomies. The calculator variables will be selected based on a systematic literature review of factors influencing CO_2_-eq emissions, with life cycle assessment (LCA) considered an established and comprehensive methodology for assessing CO_2_-eq emissions. The project will generate a comprehensive quantitative overview of the environmental impact associated with key variables involved in minimally invasive surgery. This tool has the potential to facilitate tailor-made and evidence-based decision-making and promote more sustainable surgical practices.

## 1. Introduction

One of the greatest threats to public health in the 21st century is anthropogenic climate change. Healthcare is estimated to contribute a significant 4.9% of the global carbon dioxide emissions, which exceeds that of the aviation and shipping sectors [1,2]. Operating rooms generally generate 21–33% of all hospital waste and energy, making them the most resource-intensive areas within hospitals [3,4,5,6,7,8].

Recent studies show growing interest in and motivation for sustainable decision-making among surgeons [9]. However, there are a number of barriers to sustainable surgery including lack of information, lack of guidance and support, lack of engagement by surgical teams, absence of a sustainability lead and perceived cost of implementation of green surgery initiatives [9].

Despite the growing body of literature on the environmental impact of minimally invasive surgery (MIS), this has not resulted in quantitative and objective data that can be translated into impactful decision-making for different hospitals, countries, treatments, and guidelines particularly in MIS [6,10]. Life cycle assessments (LCAs) are considered the most robust method of assessing environmental impact; however, their complexity, resource requirements, and dependence on local conditions limit their broader applicability and routine use.

There is currently no comprehensive analysis in the literature which can assist the surgical healthcare community to make decisions based on quantifiable data in minimally invasive surgery. Therefore, the aim of this study is to develop and validate an accessible, user-friendly calculator to estimate CO_2_-eq emissions for minimally invasive cholecystectomy, as it is one of the most frequently performed surgical procedures worldwide. The calculator is intended to be accessible and applicable across a range of countries, hospital volumes, and healthcare settings, subject to confirmation during external validation. This quantitative assessment could support understanding of MIS-associated CO_2_-eq emissions, national and international benchmarking, research standardization, progress monitoring, and hospital-specific advice on potential improvements.

## 2. Materials and Methods

**Figure 1:**
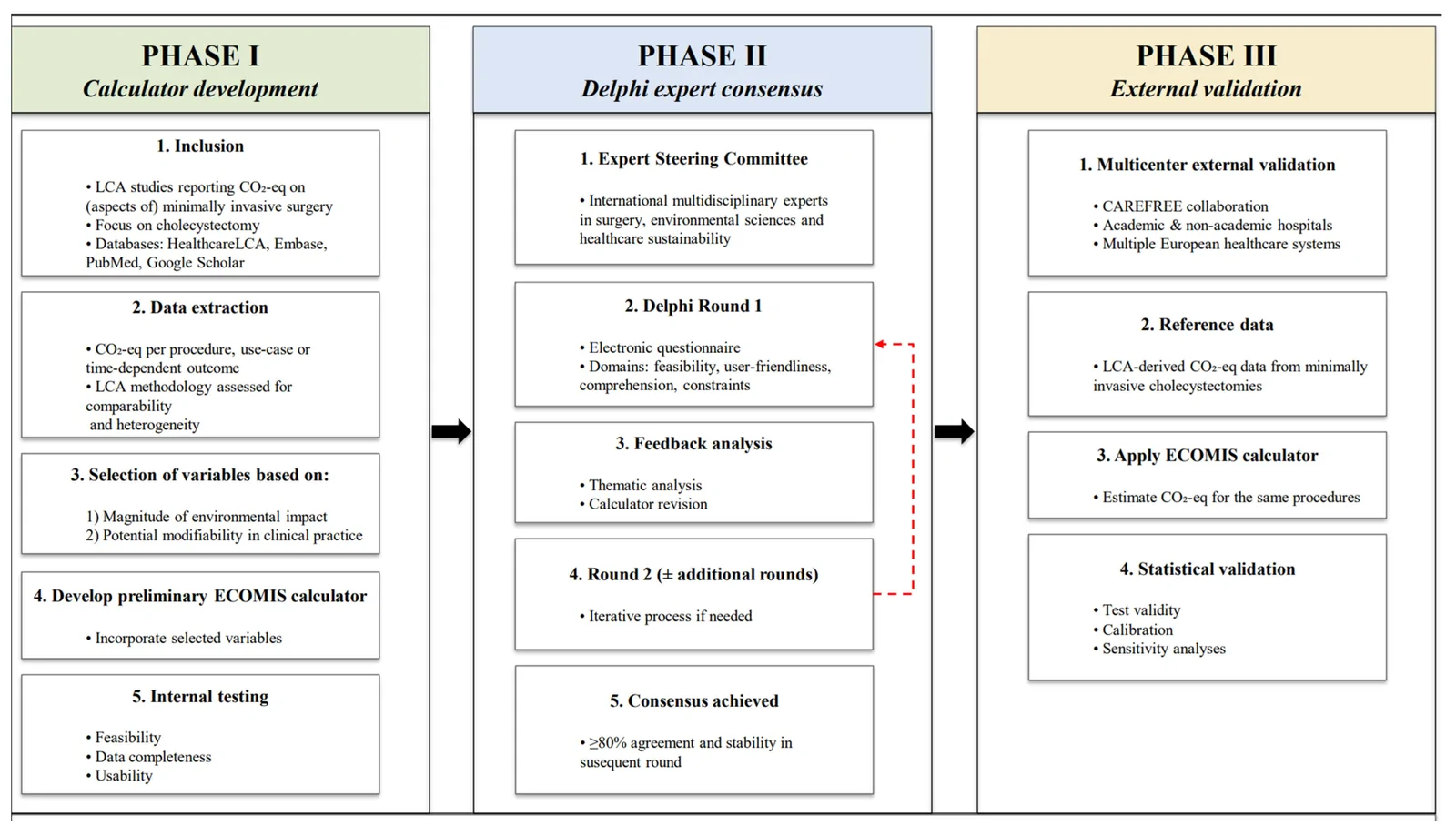
Schematic overview of project phases. ECOMIS, Environmental Impact Calculator for Minimally Invasive Surgery; LCA, life cycle assessment; CO_2_-eq, carbon dioxide equivalents; CAREFREE, Creating A healthieR Environment for FutuRE patiEnts.

This study will follow a multiphase, stepwise approach to develop a sustainability calculator for minimally invasive cholecystectomy (MIC). The calculator is part of the Environmental Impact Calculator for Minimally Invasive Surgery (ECOMIS) project. The study protocol was developed in accordance with the PRISMA-P guidelines for literature reviews, ACCORD guidelines for consensus studies, and SPIRIT-Outcomes guidelines for reporting study outcomes [11,12,13]. The present study consists of three main phases: calculator development, expert Delphi consensus survey, and external validation (Figure 1). Substantial amendments to the systematic review protocol will be documented, including their date and rationale.

### 2.1. Phase I: Literature Review and Variable Selection

A systematic literature review will be conducted to identify factors contributing to the CO_2_-eq of minimally invasive surgery. The review will investigate which procedural, equipment-related, and organizational factors contribute to CO_2_-eq emissions in minimally invasive surgery and how these contributions vary across healthcare settings. The systematic review protocol has not been prospectively registered. The review will follow the Preferred Reporting Items for Systematic Review and Meta-Analysis Protocols (PRISMA-P) guidelines [14]. The primary search will be performed in the Healthcare Life Cycle Assessment (HealthcareLCA) database, a comprehensive and globally recognized repository for LCA studies in healthcare [15].

A complementary literature review will be performed in Embase, PubMed and Google Scholar to identify potentially relevant studies not indexed in the HealthcareLCA database. The complementary strategies will combine controlled vocabulary and free-text terms relating to life cycle assessment, carbon footprinting, greenhouse gas emissions and carbon dioxide equivalents with terms for laparoscopic cholecystectomy, (minimally invasive) surgery, healthcare and operating-theatre practice. A targeted cholecystectomy search will be supplemented by broader LCA searches covering surgery, healthcare and perioperative care. Detailed contributors, including anesthesia, instruments, reusable and single-use equipment, sterilization, waste, energy, transport and operative processes, will be categorized during screening and data extraction rather than used as separate search concepts. The full search strategy will be provided as supplementary material (see Supplementary Part S1).

After deduplication, titles and abstracts will be screened independently by two reviewers, followed by independent full-text assessment of potentially eligible studies. Disagreements will be resolved by consensus or, if necessary, by consultation with a third reviewer. Data extraction will be performed independently by two reviewers using a standardized form. Records retrieved from all databases will be exported to reference management software for deduplication and screening. Screening decisions and extracted data will be documented using standardized electronic forms to ensure transparency and reproducibility. The methodological quality and reporting transparency of included LCA studies will be assessed independently by two reviewers using a predefined LCA-specific appraisal framework covering functional units, system boundaries, data sources, allocation methods, assumptions, and uncertainty analysis. Any disagreements will be resolved by consensus.

The database searches will be conducted in November–December 2026, covering studies published from 1 January 2000 up to 31 October 2026. Inclusion criteria comprise original studies performing an LCA of healthcare-related interventions, procedures, logistics, or equipment, reporting greenhouse gas emissions as kilograms of carbon dioxide equivalents (CO_2_-eq). For the purposes of this study, the calculator outcome is defined as CO_2_-eq emissions [5]. Studies focusing on cholecystectomy-related procedures or equipment will be prioritized. Reference lists and forward citations of eligible studies will also be examined. Exclusion criteria include LCAs of non-medical interventions, non-human studies, studies lacking a clear LCA methodology, or studies not reporting emissions per defined unit (e.g., per procedure or per use case). Only peer-reviewed full-text articles published in English will be included. Conference abstracts and unpublished studies will be excluded. The primary outcome will be CO_2_-eq emissions per procedure or defined use-case. Secondary outcomes will include the contributions of individual emission sources and their potential modifiability in clinical practice.

LCA results depend on methodological and contextual factors, including system boundaries, energy sources, waste management, transport, and sterilization processes [16]. These differences will be considered when assessing study comparability and selecting variables for the calculator. Variables contributing to CO_2_-eq emissions will be extracted and evaluated according to their environmental impact and potential modifiability in clinical practice, with both criteria given equal importance. The ECOMIS study team will select the preliminary variables by consensus, retaining high-impact but less modifiable factors relevant for benchmarking. The selection will subsequently be reviewed during the Delphi process.

LCA methodology will be extracted to assess comparability and heterogeneity between articles. Where feasible, environmental impact data will be harmonized to a common reporting unit, preferably kg CO_2_-eq per procedure or per defined use-case. Methodological characteristics including system boundaries, functional units, energy sources, waste treatment, transport assumptions, and sterilization processes will be extracted and used to assess comparability between studies. Results from LCAs with substantially different or non-comparable methodological frameworks will not be directly pooled or averaged, but will be considered separately during variable selection and, where applicable, in sensitivity analyses. A narrative synthesis will summarize the identified emission contributors and CO_2_-eq values. Quantitative comparisons will be limited to methodologically comparable LCA studies. Potential publication and reporting bias will be assessed qualitatively. Confidence in the evidence will be assessed qualitatively based on methodological quality, consistency, and comparability across LCA studies. To balance comprehensiveness and usability, approximately ten of the most relevant variables will be selected as an initial target for inclusion in the sustainability calculator. The final number of variables may be adjusted following internal testing and Delphi consensus. A preliminary version of the calculator will be developed by incorporating these variables. Internal testing will be conducted in five hospitals affiliated with the ECOMIS Research Sandpit group, using approximately 50 laparoscopic cholecystectomy cases (10 per center). As reference LCA data are unavailable from these centers, testing will focus on the feasibility of collecting the selected variables, data completeness, and usability. Feedback will be used to refine the calculator before Delphi consensus. Formal accuracy validation will be performed independently using CAREFREE LCA data in Phase III.

### 2.2. Phase II: Delphi Study Within the ECOMIS Expert Steering Committee

Phase II aims to optimize the applicability, usability, and clinical relevance of the calculator through expert consensus, following the ACCORD and CREDES recommendations for consensus studies [12]. A modified Delphi methodology will be used to ensure anonymity and iterative feedback [17]. The process will consist of two predefined electronic survey rounds, with the possibility of up to two additional rounds if consensus is not achieved. Surveys will be administered using Castor Electronic Data Capture (EDC), and all responses will be collected anonymously.

An international expert Steering Committee will be convened, consisting of recognized experts in hepatopancreatobiliary surgery, general surgery, environmental sciences, and healthcare sustainability. Experts will be recruited through professional networks within the European Association for Endoscopic Surgery (EAES) and the Society of American Gastrointestinal and Endoscopic Surgeons (SAGES). Invitations will be issued by the ECOMIS study team.

The Delphi panel aims to include 25 international experts representing general and minimally invasive surgery, anesthesiology, life cycle assessment, environmental sciences, and healthcare sustainability, with geographical diversity across participating countries. Eligible participants will have demonstrated clinical, scientific, or professional expertise in these fields, as evidenced by relevant publications, research involvement, or professional experience. An initial response rate of at least 70% will be targeted. Participants completing the first round will be invited to subsequent rounds, with attrition documented at each stage. Anonymized aggregated results and feedback will be provided between rounds. Experts may propose additional items during the first round, which will be reviewed by the ECOMIS study team for potential inclusion in subsequent rounds.

The Delphi questionnaire will be structured across four predefined domains: (1) feasibility, (2) user-friendliness, (3) comprehension, and (4) constraints. Depending on item characteristics, responses will include five-point Likert scales (ranging from strongly agree to strongly disagree), dichotomous responses (agree/disagree), numerical inputs, and free-text comments. The questionnaire will be developed in English. The complete survey tool is provided in Appendix A.

After each round, quantitative responses and free-text comments will be reviewed and thematically analyzed by the ECOMIS study team. Suggested modifications will be incorporated into subsequent rounds where appropriate.

Consensus will be defined a priori as ≥80% agreement among participating experts, a threshold commonly applied in Delphi studies [17]. To ensure result stability, items reaching consensus must retain consensus in a subsequent round following any revisions.

Cronbach’s alpha (α) will be calculated, where appropriate, to assess the internal consistency of multi-item domains. It will not be used as a measure of Delphi consensus. Likert-scale responses will be summarized using frequencies, percentages, medians, and interquartile ranges (IQR). Box plots will be used to visualize the distribution of Likert-scale responses across Delphi rounds. All analyses will be performed using R (version 3.6.1 or higher).

### 2.3. Phase III: External Validation of the Calculator

Phase III will consist of a multicenter external validation study to assess the generalizability, accuracy, and clinical utility of the calculator. Participating centers will include academic and non-academic hospitals performing minimally invasive cholecystectomy across different healthcare systems. For the external validation, the ECOMIS project will collaborate with the Creating A healthieR Environment for FutuRE patiEnts (CAREFREE) project [18,19].

The CAREFREE project currently investigates the carbon footprint of minimally invasive cholecystectomies in various European centers using LCA. CAREFREE uses a standardized LCA methodology across participating centers, with common functional units and predefined system boundaries. Center-specific factors will be considered when interpreting agreement between calculator estimates and reference LCA values. Criterion validity will be assessed by comparing calculator-estimated emissions with reference LCA-derived emissions. Calibration will be an assessment of systematic over- or underestimation across emission ranges.

External validation will use independent data from the multicenter CAREFREE project. All eligible laparoscopic cholecystectomy cases with available LCA data will be included. The final number of hospitals and procedures will depend on data availability. Agreement between calculator estimates and reference LCA values will be assessed using a two-way mixed-effects, absolute-agreement intraclass correlation coefficient (ICC) and Bland–Altman analysis, including mean differences and 95% limits of agreement. Sample adequacy will be evaluated based on the precision of the ICC and Bland–Altman estimates, assessed through the width of their 95% confidence intervals, as well as the number and distribution of procedures across participating hospitals. Bland–Altman results will be interpreted against acceptable error margins defined before examining the validation results, considering the intended use of the calculator and the uncertainty of reference LCA estimates. Limited precision or insufficient representation of participating centers will be acknowledged as limitations.

Calibration will be evaluated using regression analysis and calibration plots. Where sufficient data are available, exploratory analyses will assess calculator performance across different healthcare settings included in the CAREFREE project. Acceptable error thresholds will be defined before examining the validation results. Missing data will be reported as counts and percentages by variable and participating center. Validation will primarily be based on complete cases. The extent and distribution of missing data will be assessed to determine whether additional sensitivity analyses are warranted. The study will use existing CAREFREE data, and the original data collection method will be reported.

### 2.4. Storage and Access

The sustainability calculator will be hosted on a dedicated website, which has been purchased for a 5-year access period using funds from the EAES grant. The website will be secured and regularly maintained to ensure accessibility and functionality during the study and beyond. The ECOMIS research team will retain ownership and responsibility for the calculator, including its maintenance, governance, and periodic updates of emission factors. Emission factors will be derived from the LCA studies identified during Phase I. A standardized set of emission factors will be used to ensure comparability between hospitals. However, geographical and institutional differences, including variations in healthcare infrastructure, resource availability, energy sources, sterilization practices, and waste management, may influence the environmental impact of surgical procedures. Therefore, comparisons between hospitals, particularly across different healthcare systems, will be interpreted with caution. Where sufficient data are available, exploratory analyses will assess the influence of geographical and institutional characteristics on LCA outcomes and identify potential variables for future incorporation into the calculator to improve its applicability across different healthcare settings. Emission factors will be reviewed periodically and updated when new relevant evidence becomes available. Continued website maintenance beyond the initial five-year funding period will depend on available resources. All data entered into the calculator will be stored on secure servers in compliance with relevant data protection regulations, ensuring privacy and confidentiality for all involved participants. The calculator will collect only non-identifiable procedural and institutional information; no patient-identifying data will be required.

### 2.5. Stakeholders/Users

The primary users of the ECOMIS calculator include surgeons, anesthetists, and operating theatre teams performing minimally invasive cholecystectomy. Secondary stakeholders include hospital administrators, sustainability leads, and procurement teams who may use the calculator for benchmarking and quality improvement.

## 3. Expected Outcomes

The ECOMIS project aims to develop and validate a user-friendly calculator for estimating procedure-related CO_2_-eq emissions associated with laparoscopic cholecystectomy, using key variables such as anesthesia type, waste generation, instrument use (single-use vs. reusable), and operative time.

A major anticipated outcome is the establishment of benchmark values for the CO_2_-eq emissions associated with minimally invasive surgery, starting with laparoscopic cholecystectomy. By creating a standardized framework for CO_2_-eq analysis, the calculator will help institutions identify areas where sustainable practices can be implemented and improved, ultimately guiding hospital policies and procurement strategies toward more environmentally friendly and sustainable choices.

Internal testing will assess the feasibility of collecting the selected variables, data completeness, and usability. Subsequent external validation using CAREFREE data will evaluate the calculator’s accuracy and performance across different healthcare settings, helping to identify potential context-dependent limitations.

In addition to providing a quantitative assessment, the ECOMIS calculator aims to identify modifiable factors that contribute to the environmental impact of surgery. By analyzing key contributors such as anesthetic gas choices, disposable instrument usage, and waste management practices, the calculator will offer data-driven insights into areas where CO_2_-eq emissions can be reduced. This will enable hospitals to implement targeted interventions, such as increasing the use of reusable instruments, optimizing anesthesia protocols, and improving surgical waste management.

In the long term, the calculator is expected to be integrated into routine surgical practice, providing an accessible, web-based platform for ongoing environmental assessments. By making the calculator available for widespread use, the study aims to contribute to broader sustainability efforts within surgery. Additionally, the framework established through this project could be expanded to evaluate the environmental impact of other minimally invasive procedures. Ultimately, the ECOMIS calculator has the potential to influence institutional policies, surgical education, and industry standards, promoting a more sustainable approach to surgical care.

## 4. Discussion

The ECOMIS project addresses the current lack of quantitative and objective data that can be translated into impactful decision-making for different hospitals, countries, treatments, and guidelines in minimally invasive surgery. Although the environmental impact of surgery has been increasingly investigated, differences in healthcare system income, procedural volume, institutional protocols, waste-processing logistics, and general logistics make it difficult to standardize decision-making or offer a universal step-by-step approach to reducing the environmental impact of minimally invasive surgery [6,9,10].

The context-specific nature of sustainable surgery is also reflected in the different priorities reported across healthcare systems. The same study identified different priorities in low-income and high-income countries [9]. The top three priorities in low-income countries involve introduction of reusable surgical devices, reduction of consumables and reduction of use of anesthetic gases, while the high-income countries prioritized recycling, reduction of anesthesia waste and reuse of clinical waste [20]. These differences support the need for a tool that can be applied across different countries, hospital volumes, and hospital income settings, while still allowing for locally relevant interpretation.

The current and most easily implemented efforts in environmental sustainability are mostly aimed at waste recycling in operating rooms. Previous studies have reported that up to 30% of waste may be recyclable and that recycling may contribute to reducing the carbon footprint by 6% [21]. However, recycling scores low in the 10R impact ladder, since its effect on reducing the production of new products is only marginal [22]. The ECOMIS calculator may therefore help distinguish between highly visible sustainability measures and interventions with greater potential to reduce resource use, waste, and CO_2_-eq emissions.

LCA is widely used as a comprehensive methodology for assessing environmental impacts and CO_2_-eq emissions. The most methodologically complete LCA involves analysis of the cradle-to-grave impact of individual items, including raw material extraction and production, transport, use, and end-of-life processes such as cleaning, sterilization, and waste disposal [3,16]. However, LCAs are highly context-dependent (source of energy, waste processing, and efficacy of hospital logistics), thus hindering adequate comparison. Moreover, LCAs are time-consuming, resource-intensive and costly, limiting reproducibility and broader applicability. The ECOMIS calculator is therefore intended to provide an accessible and broadly applicable assessment of environmental impact rather than to replace a comprehensive institution-specific LCA. It is intended primarily as a practical benchmarking and decision-support tool. It will provide a standardized estimate of procedure-related CO_2_-eq based on a limited number of selected variables and should not be interpreted as an exact carbon footprint of an individual surgical procedure. By integrating the most relevant and potentially modifiable variables, the calculator could support sustainable decision-making and suggest environmental improvements that may reduce costs, resource use, and waste without compromising healthcare outcomes.

A major strength of the ECOMIS project is its multiphase, stepwise design. The combination of literature-based variable selection, internal testing, international expert consensus, and multicenter external validation is expected to improve the applicability, usability, accuracy, and clinical relevance of the calculator. Limiting the tool to approximately ten relevant variables may help maintain usability and accessibility, although the final number will depend on feasibility testing and Delphi consensus.

The study has several limitations. First, the reliability of the calculator will depend on the quality, comparability, and transparency of the underlying LCA literature. Existing evidence shows substantial heterogeneity in system boundaries, functional units, assumptions, and reporting methods. Second, restricting the calculator to a limited number of variables may improve accessibility but reduce completeness. Third, emission factors may change over time as energy production, manufacturing processes, sterilization technologies, and waste-management systems evolve. A standardized set of emission factors will be used across centers to ensure comparability and will be reviewed periodically. Geographical variability will be acknowledged as a limitation. Regular updating and recalibration will therefore be required. The ECOMIS calculator should be interpreted as a decision-support and benchmarking tool rather than as a source of prescriptive recommendations. Any sustainability-oriented modification of surgical practice should remain compatible with patient safety, infection prevention, clinical effectiveness, regulatory requirements, and economic feasibility.

## 5. Conclusions

The ECOMIS project provides a structured, multiphase framework for the development and validation of an environmental impact calculator for minimally invasive cholecystectomy. By integrating literature-based variable selection, expert consensus, internal testing, and multicenter external validation, the calculator aims to provide an accessible and standardized method for estimating procedure-related CO_2_-eq emissions. The proposed tool may support benchmarking, identification of modifiable environmental contributors, and tailor-made, evidence-based decision-making in surgical practice. Following successful validation, the ECOMIS framework may also provide a basis for environmental assessment of other minimally invasive procedures. Whether implementation of the calculator leads to changes in clinical practice or measurable reductions in CO_2_-eq emissions will require further evaluation.

## Data Availability

The datasets generated and/or analyzed during the current study will not be publicly available, as they include expert opinions obtained during the Delphi consensus process. However, the dataset underlying the environmental impact calculator will be made available by the corresponding author upon reasonable request. The calculator tool will be made publicly available on a website.

## Supplementary Materials

The following supporting information can be downloaded at: https://www.mdpi.com/article/10.3390/healthcare14213622/s1: Supplementary Part S1. ECOMIS Search Strategy; Supplementary Part S2. PRISMA-P 2015 Checklist.

## Abbreviations

The following abbreviations are used in this manuscript:

CAREFREE	Creating A healthieR Environment for FutuRE patiEnts project
CO_2_-eq	Carbon dioxide equivalents
EAES	European Association for Endoscopic Surgery
ECOMIS	Environmental Impact Calculator for Minimally Invasive Surgery
EDC	Electronic Data Capture
HealthcareLCA	Healthcare Life Cycle Assessment
IQR	Interquartile range
LCA	Life Cycle Assessment
MIC	Minimally invasive cholecystectomy
PRISMA-P	Preferred Reporting Items for Systematic review and Meta-Analysis Protocols
SAGES	Society of American Gastrointestinal and Endoscopic Surgeons

## Appendix A. ECOMIS Delphi—Questionnaire

DOMAIN 1—Feasibility

1. The calculator is easy to use in its current format. (1–5 Likert)
2. The data required are easily obtainable in a typical OR workflow. (1–5 Likert)
3. The calculator is feasible for use across various hospital settings, including low-resource environments. (1–5 Likert)
4. Are there any settings where using the calculator is not feasible? (Free text)

DOMAIN 2—User-friendliness

1. Completing the calculator would take an acceptable amount of time. (1–5 Likert)
2. How long did it take to complete the calculator? (Time in minutes)

DOMAIN 3—Comprehension

1. The ECOMIS calculator measures the key contributors to the CO_2_-eq of minimally invasive cholecystectomy. (1–5 Likert)
2. The calculator covers the most relevant domains of environmental impact (anesthesia, instruments, waste, operative time). (1–5 Likert)
3. The environmental impact variables selected from the literature are appropriately selected based on changeability. (1–5 Likert)
4. The output of the calculator (CO_2_-eq) is interpretable. (1–5 Likert)
5. The calculator can realistically support sustainability initiatives in operating theatres. (1–5 Likert)
6. The calculator should remain globally standardized rather than customized per country. (1–5 Likert)

DOMAIN 4—Constraints

1. What is one thing you would change or improve in the calculator? (Free text)
2. What is one thing you think absolutely must stay the same? (Free text)
3. Do you foresee any barriers in implementing/using this calculator in your institution? (Free text)
4. Any additional comments? (Free text)
