# Supplementary material for: Development of an Environmental Impact Calculator for Minimally Invasive Cholecystectomy: Protocol and Methodological Framework of the ECOMIS Project
Source: Healthcare (Basel). 2026 Oct 26;14(21):3622. doi: 10.3390/healthcare14213622 (PMC13762518; doi:10.3390/healthcare14213622)
Supplement: Supplementary file 1 [file healthcare-14-03622-s001.zip › healthcare-4532202-supplementary.pdf]

Supplementary Part S1

Full literature search strategies

Development of an environmental impact calculator for minimally invasive cholecystectomies (ECOMIS)

**Protocol version**	Version 1.7, 28 July 2026
**Eligibility period**	1 January 2000 to 1 August 2026 inclusive
**Primary source**	Healthcare Life Cycle Assessment database (HealthcareLCA)
**Complementary sources**	PubMed, Embase and Google Scholar

1. Scope and eligibility

The search is designed to identify factors contributing to carbon dioxide equivalent emissions from minimally invasive cholecystectomy. Direct cholecystectomy evidence will be prioritised, with a broader healthcare and perioperative search used to identify transferable life cycle assessment evidence.

Eligible studies will be original healthcare life cycle assessments or transparent life cycle carbon-footprint studies reporting greenhouse gas emissions as kilograms of carbon dioxide equivalents (kg CO2-eq) for a defined functional unit. Studies will be excluded if they evaluate non-medical interventions, involve non-human subjects, lack a clear life cycle methodology, or do not report emissions per defined unit.

The database strategies use two essential concepts wherever possible: an LCA or carbon-emissions concept and a procedure or healthcare concept. Detailed calculator variables, such as anaesthesia, instruments, sterilisation, waste, energy and transport, will be classified during screening and data extraction rather than required as a separate search block.

2. Search execution summary

Information source	Platform/interface	Dates searched
HealthcareLCA	Public interface	01 Jan 2000 to 01 Aug 2026
PubMed	NCBI PubMed	01 Jan 2000 to 01 Aug 2026
Embase	Embase.com	01 Jan 2000 to 01 Aug 2026
Google Scholar	Google Scholar	01 Jan 2000 to 01 Aug 2026

3. HealthcareLCA database

HealthcareLCA will be searched as the primary source. Search terms should be entered individually, without restricting country or assessment scale. Relevant surgery, procedure, equipment and service filters may be used as supplementary checks. The active filters and number of records retrieved should be recorded for each term.

Search group	Terms entered individually
Direct procedure	laparoscopic cholecystectomy; cholecystectomy
Broader procedure	laparoscopy; minimally invasive surgery; operating room; operating theatre
Equipment and processes	surgical instruments; reusable; disposable; anaesthesia; anesthesia; sterilisation; sterilization; surgical waste; energy; transport

4. PubMed

The following strategies are written for NCBI PubMed. Search 1 identifies direct cholecystectomy evidence. Search 2 identifies broader healthcare and perioperative LCA evidence. No language or article-type filters will be applied at the search stage.

Search 1. Direct cholecystectomy evidence

```
(
  "Cholecystectomy, Laparoscopic"[Mesh]
  OR cholecystectom*[Title/Abstract]
  OR "gallbladder surgery"[Title/Abstract]
)
AND
(
  "Carbon Footprint"[Mesh]
  OR "life cycle assessment"[Title/Abstract]
  OR "life-cycle assessment"[Title/Abstract]
  OR "life cycle analysis"[Title/Abstract]
  OR "carbon footprint"[Title/Abstract]
  OR "carbon dioxide equivalent"[Title/Abstract]
  OR CO2e[Title/Abstract]
  OR "CO2-eq"[Title/Abstract]
  OR "greenhouse gas emission"[Title/Abstract]
  OR "greenhouse gas emissions"[Title/Abstract]
  OR "environmental footprint"[Title/Abstract]
  OR "cradle-to-grave"[Title/Abstract]
)
AND
("2000/01/01"[Date - Publication] : "2026/04/01"[Date - Publication])
```

Search 2. Broader healthcare and perioperative LCA evidence

```
(
  "Carbon Footprint"[Mesh]
  OR "life cycle assessment"[Title/Abstract]
  OR "life-cycle assessment"[Title/Abstract]
  OR "life cycle analysis"[Title/Abstract]
  OR "carbon footprint"[Title/Abstract]
  OR "carbon dioxide equivalent"[Title/Abstract]
  OR CO2e[Title/Abstract]
  OR "CO2-eq"[Title/Abstract]
  OR "greenhouse gas emission"[Title/Abstract]
  OR "greenhouse gas emissions"[Title/Abstract]
  OR "environmental footprint"[Title/Abstract]
  OR "cradle-to-grave"[Title/Abstract]
)
AND
(
  "Surgical Procedures, Operative"[Mesh]
  OR "Operating Rooms"[Mesh]
  OR "Hospitals"[Mesh]
  OR healthcare[Title/Abstract]
  OR "health care"[Title/Abstract]
  OR hospital*[Title/Abstract]
  OR surg*[Title/Abstract]
  OR laparoscop*[Title/Abstract]
  OR "minimally invasive surgery"[Title/Abstract]
  OR "operating room"[Title/Abstract]
  OR "operating rooms"[Title/Abstract]
  OR "operating theatre"[Title/Abstract]
  OR "operating theatres"[Title/Abstract]
)
AND
("2000/01/01"[Date - Publication] : "2026/04/01"[Date - Publication])
```

5. Embase

The following strategy is written for Elsevier Embase.com using Emtree terms and title, abstract and keyword fields. The strategy should be translated if Ovid Embase is used, and the final appendix should report the platform and exact executed history.

Search 1. Direct cholecystectomy evidence

```
#1
'laparoscopic cholecystectomy'/exp
OR cholecystectom*:ti,ab,kw
OR 'gallbladder surgery':ti,ab,kw

#2
'life cycle assessment':ti,ab,kw
OR 'life-cycle assessment':ti,ab,kw
OR 'life cycle analysis':ti,ab,kw
OR 'carbon footprint':ti,ab,kw
OR 'carbon dioxide equivalent':ti,ab,kw
OR co2e:ti,ab,kw
OR 'co2-eq':ti,ab,kw
OR 'greenhouse gas emission':ti,ab,kw
OR 'greenhouse gas emissions':ti,ab,kw
OR 'environmental footprint':ti,ab,kw
OR 'cradle-to-grave':ti,ab,kw

#3
#1 AND #2
```

Search 2. Broader healthcare and perioperative LCA evidence

```
#4
'surgery'/exp
OR 'laparoscopy'/exp
OR 'operating room'/exp
OR 'hospital'/exp
OR healthcare:ti,ab,kw
OR 'health care':ti,ab,kw
OR hospital*:ti,ab,kw
OR surg*:ti,ab,kw
OR laparoscop*:ti,ab,kw
OR 'minimally invasive surgery':ti,ab,kw
OR 'operating room':ti,ab,kw
OR 'operating rooms':ti,ab,kw
OR 'operating theatre':ti,ab,kw
OR 'operating theatres':ti,ab,kw

#5
#2 AND #4

#6
#3 OR #5

Apply publication date: 01 January 2000 to 01 April 2026 inclusive.
```

6. Google Scholar

Google Scholar will be used as a supplementary source and for citation chasing. Shorter queries will be run separately because long Boolean strings are not consistently reproduced. A custom year range of 2000 to 2026 will be applied, and records published after 1 August 2026 will be excluded manually. The first 200 results per query will be screened in relevance order, unless no potentially eligible records are identified in the preceding 50 results.

No.	Query
1	"laparoscopic cholecystectomy" "life cycle assessment"
2	"laparoscopic cholecystectomy" "carbon footprint"
3	cholecystectomy reusable disposable carbon
4	"minimally invasive surgery" "life cycle assessment"
5	"operating room" "life cycle assessment"
6	"surgical instruments" "life cycle assessment"

7. Supplementary citation searching and record management

- Backward-reference checking will be undertaken for all included cholecystectomy studies and relevant systematic reviews.

- Forward-citation searching will be undertaken using the platform available to the study team, with the platform and number of records recorded.
- Records will be exported to the agreed reference manager or screening platform and deduplicated using DOI, PMID, title, author and publication year.
- Database-specific counts, duplicate counts, full-text exclusions and final included-study counts will be retained for the PRISMA flow diagram.

Supplementary Part S2

PRISMA-P (Preferred Reporting Items for Systematic review and Meta-Analysis Protocols) 2015 Checklist

Recommended items to address in a systematic review protocol*

Section & Topic	Item No.	Checklist Item	ECOMIS protocol
**ADMINISTRATIVE INFO**			page
Title			
Identification	1a	Identify the report as a protocol of a systematic review.	3
Update	1b	If the protocol is for an update of a previous systematic review, identify as such.	N/A
Registration			
Registration	2	If registered, provide the name of the registry (such as PROSPERO) and registration number.	3; protocol was not prospectively registered
Authors			
Contact	3a	Provide name, institutional affiliation, e-mail address of all protocol authors; provide physical mailing address of corresponding author.	1
Contributions	3b	Describe contributions of protocol authors and identify the guarantor of the review.	9
Amendments			
Amendments	4	If the protocol represents an amendment of a previously completed or published protocol, identify as such and list changes; otherwise, state plan for documenting important protocol amendments.	2
Support			
Sources	5a	Indicate sources of financial or other support for the review.	9
Sponsor	5b	Provide name for the review funder and/or sponsor.	9
Role of Sponsor or Funder	5c	Describe roles of funder(s), sponsor(s), and/or institution(s), if any, in developing the protocol.	9
**INTRODUCTION**			
Rationale			
Rationale	6	Describe the rationale for the review in the context of what is already known.	2
Objectives			
Objectives	7	Provide an explicit statement of the question(s) the review will address with reference to participants, interventions, comparators, and outcomes (PICO).	3

METHODS			
Eligibility Criteria			
Eligibility criteria	8	Specify the study characteristics (such as PICO, study design, setting, time frame) and report characteristics (such as years considered, language, publication status) to be used as criteria for eligibility for the review.	3-4
Information Sources			
Information sources	9	Describe all intended information sources (such as electronic databases, contact with study authors, trial registers or other grey literature sources) with planned dates of coverage.	3-4
Search Strategy			
Search strategy	10	Present draft of search strategy to be used for at least one electronic database, including planned limits, such that it could be repeated.	Suppl. 1
Study Records			
Data management	11a	Describe the mechanism(s) that will be used to manage records and data throughout the review.	3-4
Selection process	11b	State the process that will be used for selecting studies (such as two independent reviewers) through each phase of the review (that is, screening, eligibility and inclusion in meta-analysis).	3-4
Data collection process	11c	Describe planned method of extracting data from reports (such as piloting forms, done independently, in duplicate), any processes for obtaining and confirming data from investigators.	4
Data Items			
Data items	12	List and define all variables for which data will be sought (such as PICO items, funding sources), any pre-planned data assumptions and simplifications.	3-4
Outcomes and Prioritization			
Outcomes and prioritization	13	List and define all outcomes for which data will be sought, including prioritization of main and additional outcomes, with rationale.	4
Risk of Bias			
Risk of bias in individual studies	14	Describe anticipated methods for assessing risk of bias of individual studies, including whether this will be done at the outcome or study level, or both; state how this information will be used in data synthesis.	4

Data Synthesis			
Data synthesis	15a	Describe criteria under which study data will be quantitatively synthesised.	3-4
	15b	If data are appropriate for quantitative synthesis, describe planned summary measures, methods of handling data and methods of combining data from studies, including any planned exploration of consistency (such as I^2, Kendall's τ).	3-4
	15c	Describe any proposed additional analyses (such as sensitivity or subgroup analyses, meta-regression).	4
	15d	If quantitative synthesis is not appropriate, describe the type of summary planned.	4
Meta-bias(es)			
Meta-bias(es)	16	Specify any planned assessment of meta-bias(es) (such as publication bias across studies, selective reporting within studies).	4
Confidence in Evidence			
Confidence in cumulative evidence	17	Describe how the strength of the body of evidence will be assessed (such as GRADE).	4

*It is strongly recommended that this checklist be read in conjunction with the PRISMA-P Explanation and Elaboration for important clarification on the items. Amendments to a review protocol should be tracked and dated. The copyright for PRISMA-P (including checklist) is held by the PRISMA-P Group and is distributed under a Creative Commons Attribution Licence 4.0.

From: *Shamseer L, Moher D, Clarke M, Ghersi D, Liberati A, Petticrew M, Shekelle P, Stewart L, PRISMA-P Group. Preferred reporting items for systematic review and meta-analysis protocols (PRISMA-P) 2015: elaboration and explanation. BMJ. 2015 Jan 2;349(jan02 1):g7647.*
